# A longitudinal study investigating change in BMI z-score in primary school-aged children and the association of child BMI z-score with parent BMI

**DOI:** 10.1186/s12889-020-10001-2

**Published:** 2020-12-10

**Authors:** R. Mears, R. Salway, D. Sharp, J. P. H. Shield, R. Jago

**Affiliations:** 1 Centre for Academic Primary Care, Bristol Medical School, University of Bristol, Bristol, BS8 2PS UK; 2 Centre for Exercise Nutrition and Health Sciences, School for Policy Studies, University of Bristol, Bristol, BS8 1TZ UK; 3 NIHR Bristol Biomedical Research Centre, Nutrition Theme, University of Bristol, Bristol, BS1 2NT UK; 4 Faculty of Health Sciences, University of Bristol, Bristol, BS2 8DZ UK

**Keywords:** Obesity, Parents, Children, Public health

## Abstract

**Background:**

This paper aims to explore change in BMI z-score through childhood and the association between parent BMI and child BMI z-score. This is important to understand for the development of effective obesity interventions.

**Methods:**

Data from the longitudinal B-ProAct1v study (1837 participants) were analysed. A paired sample t-test examined changes in child BMI z-score between Year 1 and 4. Multivariable linear regression models examined the cross-sectional associations between child BMI z-score and parent BMI in Year 1 and 4. The influence of change in parental BMI between Year 1 and Year 4 on child BMI z-score in Year 4 was explored through regression analyses, adjusted for baseline BMI z-score.

**Results:**

There was a strong association between child BMI z-score at Year 1 and 4. Child mean BMI z-score score increased from 0.198 to 0.330 (*p* = < 0.005) between these timepoints. For every unit increase in parent BMI, there was an increase in child BMI z-score of 0.047 in Year 1 (*p* = < 0.005) and of 0.059 in Year 4 (*p* = < 0.005). Parental BMI change was not significantly associated with Year 4 child BMI z-score.

**Conclusion:**

The key indicator of higher child BMI at Year 4 is high BMI at Year 1. Further studies are needed to explore the impact of parental weight change on child BMI z-score and whether interventions targeted at overweight or obese parents, can improve their child’s BMI z-score.

## Background

The global prevalence of overweight and obese children has risen in the past four decades from 4% in 1975 to 18% in 2016 [1, 2].Within the 34 Organisation for Economic Cooperation and Development (OECD) countries, the UK is ranked 9th for childhood overweight prevalence [3, 4] In England, the National Child Measurement Programme (NCMP) assesses weight status in Reception (aged 4–5 years) and Year 6 (aged 10–11 years) of primary school [5] and local analyses have shown a rise in child BMI z-score between these ages [6]. Overweight children are more likely to become overweight adults [7], with the associated socioeconomic and health related consequences presenting a major public health challenge [8].

Parental obesity is a known risk factor for childhood obesity [9–13] though the literature is less clear whether there are differences in associations in weight according to parental gender and child gender [14–18]. The influence of parents on a child’s weight underpins the theoretical basis of family-based obesity interventions (where both the child and parent attend the intervention), which are viewed as an important strategy for the treatment of childhood overweight and obesity [19]. Many studies have demonstrated that parental weight loss during these programmes is correlated to a reduction in child BMI z-score [20, 21] More recently, evidence is emerging that children do not need to be in attendance at an intervention for their weight to improve and parent-only interventions (with the main aim of addressing the child’s overweight status) may be a more cost-effective method [22–26] Fewer studies simply observe the impact of change in parent BMI over time (gain or loss) on a child’s BMI z-score when no intervention is provided. This is important as it may provide justification for the development of parent-only interventions to prevent parental weight gain, with the indirect effect of preventing child weight gain. This might prove a cost-effective approach to tackling childhood obesity by precluding the need for weight measurements or engagement of an already overweight child in an intervention and the associated challenges that this brings, whilst also providing overweight and obesity prevention in normal weight children.

This study examines the BMI z-score of children within the wider Bristol area as they progress from Year 1 (aged 5–6 years) to Year 4 (aged 8–9 years) of primary school. This is novel as it provides insight into changes in BMI z-score in the time-period between NCMP measurements, where measurement of a child’s weight is not routinely obtained. The study also examines the association between parent BMI and child BMI z-score in Year 1 and Year 4 and explores whether associations between child and parent weight are specific to parental gender. This is important as the current literature presents conflicting findings. Finally, the study investigates whether changes in a parent’s BMI between Year 1 and Year 4 influence a child’s BMI z-score in Year 4. This is important in order to determine whether parent-only interventions, focused on preventing parental weight gain regardless of a child’s weight status, could be a viable alternative strategy to parent-child or parent-only interventions focused on the treatment of a child’s obesity.

## Methods

### Aim, design and setting

This study analysed child BMI z-score and parental BMI data collected as part of the B-ProAct1v study. The B-ProAct1v study [27, 28] was a longitudinal study to understand the association between physical activity and screen viewing patterns of children and parents. Children and at least 1 parent were recruited from 57 schools within the wider Bristol area in Year 1 (data collected from January 2012 to May 2013) and from 47 schools in Year 4 (data collected from March 2015 to July 2016) of primary school. Participation in Year 1 of the study was not a requirement of recruitment to Year 4 of the study. Parents were classified as Parent 1 (main participating parent) and Parent 2 (additional participating parent).

The study collected data from 1299 children in Year 1 (aged 5–6 years) and 1223 children in Year 4 (aged 8–9 years) of primary school [28]. Data was collected in Year 1 only for 614 children, Year 4 only for 538 children and in both Year 1 and Year 4 for 685 children. There were a similar percentage of boys (*n* = 899, 49%) and girls (*n* = 938, 51%) participating in the study. For children with parental gender data available, Parent 1 was predominantly female in both Year 1 (76%) and in Year 4 (73%).

### Ethical approval

The study was approved by the School for Policy Studies Ethics and Research Committee at the University of Bristol. Written informed parental consent was obtained from all participants.

### Measures

Children’s height and weight were measured at their primary school by B-ProAct1v fieldworkers to the nearest 0.1 cm (using a Seca Leicester Stadiometer) and 0.1 kg (using Seca digital scales) respectively, after they removed shoes and outer clothing. Height and weight were used to calculate age and gender-specific standardized child body mass index (BMI) (kg/m^2^) z-score using UK reference curves [29]. Level of deprivation were derived using the Index of Multiple Deprivation (IMD) (http://data.gov.uk/dataset/index-of-multiple-deprivation) based on participants’ home postcode. Parents reported their height and weight, gender and the gender of their participating child through a questionnaire.

### Statistical analyses

Participant characteristics are reported as follows; mean child BMI z-score in Year 1 and Year 4, mean IMD score in Year 1 and Year 4, mean age of parent in Year 1 and 4, mean BMI of parent 1 in Year 1 and Year 4 and finally the mean change in BMI of parent 1 between Year 1 and Year 4. A paired sample t-test was used to determine whether there was a change in child BMI z-score between Year 1 and Year 4 of primary school. Multivariable linear regression models were used to examine the cross-sectional associations between child BMI z-score and Parent 1 BMI in Year 1 and Year 4. Finally, the influence of change in parental BMI between Year 1 and Year 4 on child BMI z-score in Year 4 was explored through regression analyses, adjusted for Year 1 BMI z-score. This analysis was restricted to those cases where the same parent participated and provided a BMI score as ‘Parent 1’ at both time points (*n* = 353).

Participants were included in a regression analysis if data were available for all the outcome and predictor variables (complete case analysis i.e. individuals with missing data were excluded from analyses). Analyses controlled for parent gender, child gender and IMD score with robust standard errors used to account for the clustering of children in schools. All analyses were conducted in Stata version 14.1 (Statacorp, College Station, TX).

## Results

Participant characteristics are summarised in Table 1. BMI z-score data were missing in 28 children in Year 1, and 6 children in Year 4. Residual plots for all models showed no departure from model assumptions.

**Table 1:** Participant Characteristics (*n* = 1837)

	Variable	Observations	Mean (95% Confidence interval)	Standard Deviation
Year 1	Child BMI z-score	1271	0.27 (0.22, 0.32)	0.95
	IMD Score	1172	14.98 (14.24, 15.72)	12.86
	BMI of parent 1	1002	25.38 (25.10, 25.66)	4.56
	Age of parent 1	1000	37.73 (37.38, 38.08)	5.70
Year 4	Child BMI z-score	1217	0.35 (0.28, 0.41)	1.07
	IMD score	1204	15.94 (15.14, 16.74)	14.07
	BMI of parent 1	950	25.92 (25.62, 26.23)	4.85
	Age of parent 1	962	41.36 (40.97, 41.76)	6.24
Year 1 to Year 4	Change in Parent 1 BMI between Year 1 and Year 4	353	0.24 (−0.03, 0.51)	2.62

The proportion of overweight or obese children in Year 1 was 19% (*n* = 245/1271) and in Year 4 was 25% (*n* = 304/1217). For children with parent 1 BMI data available, the proportion of overweight or obese parents in Year 1 was 45% (*n* = 447/1002) and in Year 4 was 49% (*n* = 470/950). Table 2 summaries the weight categories of children and parent participants in more depth.

**Table 2:** Weight categories of child and parent participants

	Year 1 Child N (% in weight category according to child gender)		Year 4 Child N (% in weight category according to child gender)		Year 1 *Parent 1* N (% in weight category according to parent gender)		Year 4 *Parent 1* N (% in weight category according to parent gender)	
	Female	Male	Female	Male	Female	Male	Female	Male
Normal or underweight	504 (81%)	522 (80%)	478 (72%)	435 (79%)	445 (59%)	110 (44%)	370 (54%)	110 (42%)
Overweight	72 (12%)	77 (12%)	88 (13%)	52 (9%)	203 (27%)	99 (40%)	203 (30%)	119 (45%)
Obese	46 (7%)	50 (8%)	98 (15%)	66 (12%)	106 (14%)	39 (16%)	113 (16%)	35 (13%)

### Change in BMI z-score between year 1 and year 4

For those children measured both in years 1 and 4, the mean BMI z-score increased from 0.198 in Year 1 to 0.330 in Year 4 (mean difference 0.132, 95% CI 0.080 to 0.183, *p* = < 0.005, *n* = 667). The mean BMI z-score for boys increased from 0.160 in Year 1 to 0.276 in Year 4 (mean difference 0.116, 95% CI 0.036 to 0.195, *p* = 0.0047, *n* = 313). The mean BMI z-score for girls increased from 0.232 in Year 1 to 0.377 in Year 4 (mean difference 0.146. 95% CI 0.079 to 0.212, *p* = < 0.005, *n* = 354).

### Cross-sectional associations between child BMI z-score and parental BMI in year 1 and year 4

For those children with BMI z-score measurements in Year 1, with associated parent 1 BMI score and IMD score recorded (*n* = 976), for every unit increase in parent 1 BMI, there was a 0.047 increase in child BMI z-score (see Table 3, 95% CI 0.031 to 0.063, *p* = < 0.005). Within this group, the mean parental BMI was 25.4 and the mean child BMI z-score was 0.235.

**Table 3:** Year 1 Cross-sectional Linear Regression of Parental BMI on Child BMI z-score

*n* = 976			
Child BMI Z-score	Coefficient	95% CI	*P*-value
BMI of Parent 1	0.047	0.031, 0.063	< 0.005
Gender of Parent 1– Female^a^	0.167	0.046, 0.288	0.008
Child Gender – Female^a^	− 0.049	- 0.170, 0.071	0.418
IMD Score	0.006	0.001, 0.011	0.025

^a^Compared to reference category male

*IMD* Index of Multiple Deprivation, *n* number of observations, *BMI* Body Mass Index

For those children with BMI z-score measurements in Year 4, with associated parent 1 BMI and IMD score recorded (*n* = 935), for every unit increase in parent 1 BMI, there was a 0.059 increase in child BMI z-score (see Table 4, 95% CI 0.044 to 0.075, *p* = < 0.005). Within this group, the mean parental BMI was 25.9 and the mean child BMI z-score was 0.321.

**Table 4:** Year 4 Cross-sectional Linear Regression of Parental BMI on Child BMI z-score

*n* = 935			
Child BMI Z-score	Coefficient	95% CI	*P*-value
BMI of Parent 1	0.059	0.044, 0.075	< 0.005
Gender of Parent 1 – Female^a^	0.100	−0.068, 0.269	0.237
Child Gender – Female^a^	0.069	−0.087, 0.225	0.380
IMD Score	0.007	0.002, 0.012	0.007

^a^Compared to reference category male

Further exploratory regression analyses demonstrated that in Year 1, for each unit increase in female parent BMI, there was a 0.052 increase in child BMI z-score (95% CI 0.037 to 0.067, *p* = < 0.005, *n* = 817) and for each unit increase in male parent BMI, there was a 0.037 increase in child BMI z-score (95% CI 0.012 to 0.063, *p* = 0.005, *n* = 492). In Year 4, for each unit increase in female parent BMI, there was a 0.057 increase in child BMI z-score (95% CI 0.039 to 0.075, *p* = < 0.005, *n* = 748) and for each unit increase in male parent BMI, there was a 0.071 increase in child BMI z-score (95% CI 0.045 to 0.096, *p* < 0.005, *n* = 468).

There was also an association between IMD score and child BMI z-score both in Year 1 and Year 4. In Year 1, for every unit increase in IMD score, there was a 0.006 increase in child BMI z-score (see Table 2, 95% CI 0.001 to 0.011, *p* = 0.025). In Year 4, for every unit increase in IMD score, there was a 0.007 increase in child BMI z-score (see Table 3, 95% CI 0.002 to 0.012, *p* = 0.007).

### Association of Child BMI z-score in year 4 with change in parental BMI between year 1 and year 4

There was no statistically significant association between change in parental BMI between Year 1 and Year 4 and Year 4 child BMI z-score, adjusted for Y1 BMI z-score (see Table 5, *p* = 0.056, 95% CI − 0.001 to 0.055, *n* = 343). There was a strong association between child BMI at Year 1 and Year 4, but no differences by child gender.

**Table 5:** Association between Child BMI z-score in Year 4 and Change in Parental BMI between Year 1 and Year 4

*n* = 343			
Child BMI z-score in Year 4	Coefficient	95% CI	*P*-value
Change in parental BMI	0.027	−0.001 to 0.055	0.056
Child BMI z-score in Year 1	0.873	0.769 to 0.978	< 0.001
Parent Gender - Female^a^	0.015	−0.116 to 0.147	0.816
Child Gender - Female^a^	−0.018	−0.159 to 0.122	0.794
IMD Score in Year 4	0.003	−0.002 to 0.008	0.192

^a^Compared to reference category male

The subsample of participants (*n* = 343) available for this analysis had a lower mean IMD score in Year 4 (13.96, 95% CI 12.59, 15.33) compared to the main sample (15.94, 95% CI 15.14, 16.74). Mean child BMI z-score in Year 4 was lower in this subsample (0.23, 95% CI 0.12, 0.34) compared to the main sample (0.35, 95% CI 0.28, 0.41). Mean BMI of Parent 1 was similar in both the subsample (Yr 1: 25.08, 95% CI 24.63, 25.3, Yr 4: 25.28, 95% CI 24.80, 25.76) and the main sample (Yr 1: 25.38, 95% CI 25.10, 25.66. Yr 4: 25.92, 95% CI 25.62, 26.23).

## Discussion

### Main findings of this study

This current study found a strong association between child BMI z-score at Year 1 and Year 4. Mean BMI z-score increased between these time points for both boys and girls. Children from more deprived areas had a higher BMI z-score than those from less deprived areas. Increased parental BMI was associated with increased child BMI z-score in both Year 1 and Year 4, though this effect was small. Further exploratory analyses demonstrated that this association was observed between both maternal BMI and child BMI z-score and paternal BMI and child BMI z-score. Finally, regression analyses showed that there was no statistically significant association between change in parental BMI between Year 1 and Year 4 and Year 4 child BMI z-score.

### Implications of findings in context of existing literature

It has not been possible to conduct analyses tracking a child’s weight status from the national NCMP database yet, as up until 2013, all local data was anonymised prior to being uploaded which has prevented data linkage [30]. Although the Health Survey for England provides data on childhood obesity prevalence, different children are selected each year thereby preventing tracking analyses [31]. Studies using local NCMP data (with participant identifiable data) have shown a rise in child BMI z-score [6] and the prevalence of obesity [32] between Reception and Year 6. Our study uses longitudinal data and is novel in providing insight into the changes occurring between NCMP measurements and determines whether child BMI z-score is already increasing by Year 4 or whether this rise takes place after Year 4.

Parental obesity is a known risk factor for childhood obesity [9–13]. The literature is less clear regarding the significance of parental gender on offspring obesity risk. Some studies suggest maternal obesity to be of greater importance to offspring (of either gender) than paternal obesity [14, 15] whereas Freeman et al. reported that having an overweight or obese father, but a healthy weight mother, increased the odds of a child becoming obese, but not the reverse scenario (i.e. having an overweight or obese mother with a healthy weight father was not a significant predictor of childhood obesity) [16]. More recently, Aris et al. have shown paternal overweight status to be a significant risk factor for childhood obesity [17]. Some studies report gender assortative relationships between parental and offspring weight status (i.e. mother-daughter and father-son associations in BMI / BMI z-score) [18] whereas other studies have found minimal or no compelling evidence of these associations [13, 33]. The Health Survey for England 2017 found child BMI status to be independently associated with both their mother’s and father’s BMI status [34]. Similarly, our study found both paternal and maternal BMI to be important predictors of a child BMI z-score in both Year 1 and Year 4 of Primary School. Higher IMD scores were also associated with higher child BMI z-scores, supporting previous findings in the literature that social deprivation increases obesity risk [34–36].

The current study observed how parental BMI change between Year 1 and Year 4 is associated with a child’s BMI z-score in Year 4. Whilst there are numerous studies reporting on correlations between parental weight loss and child BMI z-score reductions in family-based treatments for childhood obesity [20, 21], there are fewer studies simply observing the effect of parental BMI change over time on a child’s BMI z-score when no intervention is provided. This is important as it may provide justification towards allocating scarce public health funding [37] to ‘parent-based programmes’ targeted at preventing parental weight gain (even where the child is normal weight) and thereby child weight gain. This in turn may generate a viable alternative cost-effective strategy to parent-only or parent-child interventions aimed specifically at overweight or obese children. There is already emerging evidence that parent-only interventions (where only the parent attends the intervention) may be as effective [22, 23, 38] as the current gold-standard family-based interventions (where both parent and child attends) [19] for the treatment of childhood overweight/obesity,. However, there is less extensive literature looking at parent-only interventions (focused specifically on preventing parental weight gain) for the prevention and treatment of childhood obesity.

Our study showed that there was no significant association between change in parental BMI between Year 1 and Year 4 and Year 4 child BMI z-score. These findings need to be interpreted with caution as the sample size was relatively small, and missing data prevented analysis of all participants and their parents taking part in the B-ProAct1v study. An Indonesian observational study reported a father’s weight gain or weight loss to be correlated to their daughters’ weight but not their sons, whereas a mother’s weight gain or loss was correlated to both the weight of sons and daughters [39]. Further sufficiently powered observational studies are needed to explore this.

### Limitations of this study

The Year 1, B-ProAct1v child weight data (measured in 2012/2013), showed a slightly lower percentage of children classified as overweight (12%) or obese (8%) compared to Bristol NCMP data from Reception (measured in 2011/2012, i.e. the children who would be moving into Year 1 in 2012/2013) where there were 14% classified as overweight and 10% classified as obese. It is unlikely that the prevalence of obesity reduces between Reception and Year 1 and so this discrepancy may indicate consent bias in the B-ProAct1v project (i.e. overweight or obese children and their parents may have been less likely to agree to participate in the study than their normal weight counterparts).

For recruitment to the B-ProAct1v study, a child and at least one parent (designated as parent 1) needed to consent to participate in data collection. This led to the main analyses in this current study focusing on child BMI z-score and Parent 1 BMI data and limited analyses of child BMI z-score with gender of parent or child. Parent 1 was usually female, so this also limited the power of the study to detect associations between maternal and paternal BMI and child BMI z-score as the number of male parents designated as Parent 1 was considerably smaller than the number of female parents. There was limited BMI data available for the same parent 1 in Year 1 and Year 4 which restricted analyses investigating how parental change between Year 1 and Year 4 influences Year 4 child BMI z-score to a relatively small sample of Year 4 children. Due to the limited data, it was also not possible to undertake separate analyses according to parental gender or child gender.

There was no assessment of pubertal stage for children participating in the study, though this is unlikely to impact the results given that few children would be in puberty in Year 4 of school. Finally, parental BMI was calculated from self-reported height and weight data. Self-report data has previously been shown to slightly overestimate height and underestimate weight [40, 41]. In this study, this could result in underestimation of the observed associations. However, previous large validation studies have supported the accuracy of self-reported adult height and weight measurements to calculate BMI [42, 43]. Children’s height and weight were measured at their primary school by B-ProAct1v fieldworkers.

## Conclusion

This study fills the gap between NCMP data (collected in Reception and Year 6) and shows that there is a strong association between child BMI z-score at Year 1 and Year 4 with mean BMI z-score increasing between these time points for both boys and girls. We found that both the mother and father’s BMI score were independently associated with their child’s BMI z-score. Although no significant association was found between change in parental BMI between Year 1 and Year 4, and child BMI z-score in Year 4, this analysis was limited to a relatively small sample of children where parental BMI data was available at both time points. Further sufficiently powered studies, are needed to explore the impact of parental weight change on child BMI z-score and whether interventions aimed at preventing weight gain in parents are a viable alternative to parent-only or parent-child interventions aimed at overweight or obese children. However, our study demonstrates that the key indicator of higher child BMI at Year 4 is high BMI at Year 1 and so interventions should be considered at an early age.

## Data Availability

Anonymised data from this project are available on reasonable request.

## Abbreviations

BMI: Body Mass Index
CI: Confidence Interval
IMD: Index of Multiple Deprivation
NCMP: National Child Measurement Programme
OECD: Organisation for Economic Cooperation and Development (OECD) countries
